# Forecasting the global demand for HIV monitoring and diagnostic tests: A 2016-2021 analysis

**DOI:** 10.1371/journal.pone.0201341

**Published:** 2018-09-19

**Authors:** V. Habiyambere, B. Dongmo Nguimfack, L. Vojnov, N. Ford, J. Stover, L. Hasek, P. Maggiore, D. Low-Beer, M. Pérez Gonzàlez, D. Edgil, J. Williams, J. Kuritsky, S. Hargreaves, T. NeSmith

**Affiliations:** 1 Department of HIV/AIDS, WHO, Geneva, Switzerland; 2 Avenir Health, Glastonbury, Connecticut, United States of America; 3 CHAI, Boston, Massachusetts, United States of America; 4 USAID, Washington DC, United States of America; 5 US CDC, Atlanta, Georgia, United States of America; 6 International Health Unit, Imperial College London, London, United Kingdom; Boston University School of Public Health, UNITED STATES

## Abstract

**Introduction:**

Despite considerable progress, just over half of the 37 million people eligible to start antiretroviral therapy (ART) have accessed treatment and millions of HIV-positive people still do not know their status. With demand for ART continuing to grow, meeting the ambitious 90-90-90 HIV treatment targets will depend on improved access to high-quality diagnostics to both diagnose infection and monitor treatment adherence in low and middle-income countries (LMICs). Robust projections of future demand for CD4, viral load (VL), HIV early-infant-diagnosis (EID) tests and HIV rapid diagnostic tests (RDTs) are needed as scale-up continues.

**Methods:**

We estimate the current coverage for HIV diagnostics and project future demand to 2021 using a consolidated forecast using data on past coverage and current demand from a number of sources, from 130 predominantly LMIC countries.

**Results:**

We forecast that the overall number of CD4 tests is expected to decline between now and 2021 as more countries adopt test-and-treat and shift to VL testing for patient monitoring. Our consolidated forecast projects a gradual decline in demand for CD4 tests to 16.6 million by 2021. We anticipate that demand for VL tests will increase to 28.5 million by 2021, reflecting the increasing number of people who will receive ART and the adoption of VL testing for patient monitoring. We expect that the demand for EID tests will grow more rapidly than in past years, driven by the implementation of testing at birth in programmes globally, in line with WHO guideline recommendations, doubling to 2.1 million tests by 2021. Demand for rapid diagnostic tests is also likely to increase, reaching 509 million tests by 2021.

**Discussion:**

In order to achieve the ambitious 90-90-90 targets, it will be essential to maintain and improve access to CD4, VL, EID tests and RDTs. These projections provide insight into the global demand we can expect to see for these HIV monitoring and diagnostic tests, both in relation to historical trends, and the 90-90-90 targets. Our projections will better enable producers to ensure adequate supply, and to support procurement organisations in planning future funding and purchase plans to meet the anticipated demand. The findings highlight the ongoing need for governments and international funding bodies to prioritise improving capacity and access to HIV diagnostic and monitoring technologies in line with demand.

## Introduction

In low- and middle-income countries (LMICs) with the highest burden of HIV, coordinated action is now urgently needed to ensure that the ambitious 90-90-90 HIV treatment targets set out by the Joint United Nations Programme on HIV and AIDS are met [1]. The targets envision that by 2020, 90% of all people living with HIV will know their status, 90% of those diagnosed as HIV positive will start antiretroviral therapy (ART), and 90% of all people receiving ART will have durable viral suppression. Furthermore, the 2015 World Health Organization (WHO) guidelines [2] recommend that ART is prescribed to all people as soon as possible after an HIV-positive diagnosis regardless of CD4 cell count, which has and will continue to increase the number of people who need to be started and maintained on treatment. Despite the considerable progress to date, only half of all people eligible to start ART have accessed treatment: by the end of 2016, 36.7 million people were living with HIV and 19.5 million were accessing ART globally, which represents a coverage of 53% [3,4]. In addition, millions of HIV-infected people still do not know their status [5]. This is in large part due to the significant gaps in capacity, low demand for HIV tests resulting in low service utilisation, and low access to diagnostic tests [6,7,8]. Indeed, with demand for ART continuing to grow, achieving the 90-90-90 targets will undoubtedly be dependent on service providers focusing their efforts to higher-prevalence groups/districts and improved access to high-quality diagnostics to both diagnose and monitor the HIV infection.

Running effective treatment programmes requires high-quality HIV testing technologies, including CD4 testing to assess the level of immunosuppression and risk of opportunistic diseases, viral load (VL) testing to monitor treatment efficacy, early infant diagnosis testing (EID) to determine HIV-infection status in HIV-exposed children born to women living with HIV, and other diagnostic capabilities within a tiered laboratory network as recommended in the Maputo Declaration [9]. HIV rapid diagnostic tests are additionally needed to improve access to HIV diagnosis in LMIC settings. More than 600 million adults were screened with the rapid HIV test in 120 LMICs between 2010 and 2014 [10] and many rapid diagnostic tests for HIV such as HIV self-testing technologies are now on the market and will undoubtedly make testing more efficient [11,12]. Several point of care testing technologies are available and in development for the measurement of CD4 cell count and HIV viral load that have promise to further extend access to HIV monitoring tests [13].

Availability of CD4 testing is increasing in LMICs, and in many countries there are a sufficient number of instruments to meet current demand [6,8]. While it is anticipated that a shift towards starting ART irrespective of CD4 cell count and using VL alone for routine monitoring will decrease the demand for CD4 tests [11,10], CD4 cell count will remain for many developing countries which have no test-and-treat policy, an important part of HIV treatment and care, particularly to support decision-making around ART initiation, clinical management, and treatment monitoring [14,15]. In addition, in many countries access to VL monitoring remains limited [16], and in these settings CD4 cell count remains important for monitoring response to treatment. Whilst CD4 and VL testing instruments may be available in many LMICs, there is significant underutilisation of these technologies: in 2013, for example, only 14% of existing CD4 capacity and only 37% of VL capacity were utilised [6].

WHO recommends that all LMICs phase in VL monitoring, testing all patients at 6 and 12 months after ART initiation, and then at least every 12 months [10]. This approach aims to ensure early indication of when enhanced adherence support is needed and when a person may need a treatment switch. Improving access to VL testing, the preferred way to monitor a patient’s response to ART, is critical to prolonging the use of first-line regimens and thus ensuring the longevity of treatment programmes globally by preventing drug resistance from developing [7].

The demand for EID tests is expected to increase, driven by changes in the WHO testing guidelines to include testing at birth, the introduction of point-of-care EID testing and increased donor funding. A recent WHO Survey highlights high coverage of EID testing [6,8]; however, it also highlighted key gaps in utilisation of EID and VL testing instruments, and there is further evidence that delays in obtaining results exacerbate the significant losses in the testing-to-treatment cascade, with only 30% of perinatally infected infants receiving timely ART [17].

Developments in this field of in vitro diagnostic devices (IVD) are rapid and there is a need for robust projections of future demand for RDTs, CD4, VL, and EID tests in LMICs as scale-up continues. This will mean that donor funding, implementing partners, governments and procurement organisations such as USAID, UNICEF, Global Fund, the Global Supply Chain Program, UNDP and national central medical stores, can ensure that adequate funding and resources are in place, that capacity is distributed to meet demand, and that reagent producers anticipate the demand and produce appropriate quantities of reagents. To this end we aim to estimate the current coverage for HIV monitoring and diagnostic tests and project future demand to 2021, using consolidated estimates from country-specific analyses and linear extrapolations of past trends.

## Methods

### Overall approach

In order to project future demand for HIV monitoring and diagnostic tests in LMICs through to 2021, we did a consolidated forecast using data on past coverage and current demand from a number of sources. These sources include the following: (1) WHO, the WHO antiretroviral (ARV) medicine and diagnostic use survey [6,8], (2) CHAI, projections from the Clinton Health Access Initiative (CHAI), (3) GPRM, the Global Price Reporting Mechanism of WHO, and (4) GAM, the Global AIDS Monitoring system for HIV testing and linear extrapolations of past coverage. We analysed data for 130 countries, which includes 114 LMICs and 16 high-income countries.

Data were analysed in Microsoft Excel 2016. Once data had been collated and analysed, a technical working group composed of WHO, the United Nations Children’s Fund (UNICEF), UNAIDS, the United States Center for Diseases Control and Prevention (CDC), the United States Agency for International Development (USAID), CHAI, Avenir Health, and the Global Health Supply Chain Program reviewed the results and provided feedback about data quality, assumptions for projection methodology, and the consolidation methodology of the interim results. This consultation process was then repeated involving a larger group that included diagnostic manufacturers, partners including funding agencies, country representatives, civil society and stakeholders who provided feedback that was subsequently incorporated.

### Assessment of current coverage

We estimated current total coverage in 2015 using data from the WHO ARV medicine and diagnostic use survey (WHO method) [6,8]. We extracted data from all LMICs who responded. For countries not reporting in this survey, we estimated coverage as follows. For the 70% of countries who reported data for at least two years from 2011–2016, coverage was estimated as a linear extrapolation for 2015. For the 17% of countries who only reported data for one year, we estimated coverage by multiplying need in 2015 by the ratio of coverage in the year of the report, and estimated coverage in 2015. The formula of the ratio has two values: coverage in the numerator and need in the denominator. Coverage is referred to number of tests reported by the country in a specific year. We used the number of people receiving ART to estimate the need for CD4 and VL tests, while the number of HIV-positive pregnant women was used to estimate the need for EID tests. Where countries had reported no data in any year—for these countries we estimated coverage for 2015 by multiplying need (number of people on ART) by the average ratio of CD4 or VL tests needed by reporting countries in 2015.

### Calculating future projections

Future projections for total need and anticipated demand used a consolidated approach of two methods: the CHAI method and the WHO method. From the CHAI method, future projections are based on annual CHAI HIV diagnostics forecasts, which include data through to the end of 2015 [18] (CHAI method as described below). In these forecasts developed by CHAI, demand is estimated in the following countries based on their high test volumes and high HIV burden: 21 LMICs were included in the CHAI forecasts for CD4 count and VL testing (Botswana, Brazil, Cameroon, China, Côte d’Ivoire, Ethiopia, India, Kenya, Lesotho, Malawi, Mozambique, Myanmar, Nigeria, Rwanda, South Africa, Swaziland, Uganda, United Republic of Tanzania, Viet Nam, Zambia and Zimbabwe). 26 LMICs were included for EID (Angola, Botswana, Brazil, Burundi, Cameroon, Chad, China, Cote d’Ivoire, DRC, Ethiopia, Ghana, India, Kenya, Lesotho, Malawi, Mozambique, Namibia, Nigeria, Rwanda, South Africa, Swaziland, Tanzania, Thailand, Uganda, Zambia, Zimbabwe). These countries represent 87% of the demand for CD4 count tests, 77% of the demand for viral load testing, and 96% of the demand for EID. Projections for countries not included in the CHAI analysis were made by linear extrapolation of the historical coverage from the WHO method [6,8]. Both CHAI and WHO methods were consolidated to generate the global projections of HIV monitoring and HIV diagnosis tests from 2016 to 2021. Historical data on the number of people receiving ART were obtained from the UNAIDS AIDS info database [19].

Annual growth in ART patient numbers are based on the Fast-Track projection [18]. Estimates of people in pre-ART are based on UNAIDS data of total HIV-positive people who are expected to know their status compared to those receiving ART in each country. They are further reduced based on literature on pre-ART care retention and access to CD4 testing, with the assumption that 44% of HIV-positive adults are monitored and staged using CD4 testing, but are not eligible for ART [18], and accounting for anticipated changes in ART initiation policies over time (see below).

The specific method used for CHAI forecasts for CD4 testing, EID, and VL testing, is outlined below:

#### (i) CD4 testing forecast

Estimates of historical coverage for CD4 count tests were produced using procurement data from CHAI, UNICEF, WHO, the Global Fund to Fight AIDS, Tuberculosis and Malaria and the Partnership for Supply Chain Management for 2013–2016 compared to country-reported procurement and service statistics from 2012–2015. Analyses of these procurement data were conducted for each included country. Projected demand was modelled by applying a linear growth rate to the procured country-reported testing volumes from 2015. A linear growth rate utilising available country targets for 2016–2019 was applied to the country-reported testing volumes in 2015 to model projected demand. The country targets were reported by 21 CHAI country teams during an annual data collection exercise. The targets are official national targets, approved by ministries of health (MoHs). However, not all countries had national targets established or reported for each year from 2016 to 2019. Whenever country targets were not available, CHAI used testing need according to WHO guidelines as a proxy of the country targets. Due to the lack of national targets for each year for each country, and because targets were defined differently (sometimes as national targets, sometimes as ‘testing needs’), the annual growth rate for historical testing volumes was weighted at 70% and annual growth rate for country targets were weighted at 30% in the baseline scenario.

Need was defined as the number of people expected to receive CD4 cell count testing multiplied by the number of CD4 cell count tests per person per year according to the WHO guidelines [2]. WHO recommends CD4 cell count testing for ART monitoring where VL monitoring is not yet available, and in all settings at first presentation to care (or re-engagement in care for those patients who may have interrupted treatment) to evaluating the risk of opportunistic infections, rather than to stage HIV-positive people. Since 2016, these recommendations have been gradually implemented in selected countries that have either made policy plans publicly available or will have achieved at least 75% coverage of ART. The theoretical need for CD4 testing follows the scaling up of VL testing and shift to a test-and-treat policy as countries reach ART coverage of HIV-positive people of 75% or more. In the CHAI projection, the CD4 need was estimated by multiplying one CD4 test by the number of new patients starting ART each year, two CD4 tests for all patients not covered by viral load, two CD4 tests for patients with elevated viraemia (assumed to be 16%), and two CD4 tests for every patient on pre-ART, where applicable.

#### (ii) EID testing forecast

For the EID testing forecast we used annual reported data from the 26 CHAI country teams based on their higher volume of EID tests, CHAI-UNITAID Paediatric Grant reports (2007–2011), UNAIDS data, the 2013 Global Plan, UNICEF Children and AIDS Stocktaking Reports (2007–2013), and Ministry of Health guidelines and programme reports. We used the largest figure available from all sources for historical coverage. To project the future demand for EID tests, we used a linear extrapolation based on the historical test volumes based on data from 2011–2015. However, whenever this produced estimates that were higher than the need, we used the need calculated based on WHO guidelines [10] and the number of eligible patients.

Our model assumed that testing at birth was introduced in 2016 in South Africa, 2017 in Swaziland, 2018 in Kenya and Zimbabwe, and 2019 elsewhere, in addition to the 4–6 week DNA-polymerase chain reaction (PCR) test. Our model also assumes that EID testing coverage will follow the growth of overall EID coverage from 2013 (modified in line with facility delivery rates), and that point-of-care EID test will begin in 2016 or 2017 in the countries included in this analysis.

#### (iii) VL testing forecast

We used annual reported data from the 21 CHAI country teams and historical testing volume reported publicly by certain countries (Kenya, Uganda, and Malawi). Given that VL testing is relatively less accessible for most LMICs, the CHAI forecast for VL testing differs from CD4 and EID forecast models. We utilised historical analogues and estimated demand based on predicted VL coverage in a given year multiplied by expected need for a country. The predicted coverage rate utilises the combined score of a set of index factors (status of funding and procurement, age of the routine monitoring programme, status of guidelines and policy [including adoption of WHO viral load guidelines [10], historical growth rate, and historical ratio between the number of VL tests and the total need). The scores are generated based on data from country implementation plans, procurement and supplier sales records, country-reported volumes, and global policy documents, and correspond to the predicted coverage categories (high, medium, low, and mature growth rates). We then mapped theses scores to four historical growth analogues, which estimate the number of VL tests conducted annually compared to the number of ART patients identified during scaling up of VL testing in several countries in sub-Saharan Africa, utilising country targets instead of predicted coverage estimates where they are found to be more conservative.

### Consolidated forecast

In the consolidated forecast, we have combined projections for the 21 countries for CD4 and VL tests and the 26 countries for EID tests included in the CHAI analyses, with those from the WHO method for countries not included in the CHAI analyses, in order to have the total projection for all the 130 countries.

For CD4 tests, we extrapolated the aggregate trend from 2011–2015 for countries not included in the CHAI analysis to 2021 using a logarithmic extrapolation, reflecting the anticipated decrease in CD4 test consumption.

For countries not included in the CHAI analysis with data for two or more years, the projection is a linear extrapolation of the past trend. For countries with data for only one year, demand was projected by applying the ratio of the number of VL tests reported to the number of people receiving ART to the estimated future number of people receiving ART. For countries with no data, we projected future demand for VL by multiplying the estimated number of people on ART in that country by the ratio of the total number of VL tests each year reported to the total number of people receiving ART each year.

The projection for countries not included in the CHAI analysis with data for two or more years was a linear extrapolation of the past trend. For countries with data for only a single year, demand was estimated by applying the ratio of early infant diagnosis tests and number of HIV+ women receiving services for prevention mother-to-child transmission of HIV to the estimated future number of women receiving these services. For countries with no data, we projected future demand by multiplying the estimated number of women receiving services for prevention the mother-to-child transmission of HIV by the ratio of the total number of EID tests reported each year by reporting countries divided by the total number of HIV+ pregnant women receiving these services.

For HIV rapid diagnostic tests, data were collected from country-specific reports and from the 2015 and 2016 WHO diagnostic surveys [6,8]. We estimated future demand in each country by multiplying the projected number of HIV-positive people in each future year by the ratio of number of rapid diagnostic tests reported in these years divided by number of HIV-positive people in that year. We projected the aggregate demand for RDT as a log extrapolation of demand in these two years (Test[year] = 287,581284 x 106,526,682 x ln[year– 2013]), accounting for the anticipated decrease in growth rate as an increasing proportion of HIV-positive people know their status.

### The 90-90-90 treatment target

The consolidated forecasts were compared to the Fast-Track forecasts [18]. Fast-Track is an aspirational initiative developed by UNAIDS to describe a path to eliminate AIDS as a public health threat by 2030. Fast-Track is the comprehensive response to achieve the UNAIDS goals of reducing new infections and AIDS deaths by 90% from 2010 to 2030. It includes scaling up a variety of interventions including condoms, medical male circumcision, programs for key population, PrEP, and treatment. 90-90-90 is the treatment part of Fast-Track [18]. Fast-Track envisions rapid increases in coverage of key prevention and treatment programs by 2020 and further increases by 2030. The 90-90-90 treatment target was proposed by UNAIDS in 2014 as a new target for scaling up treatment, envisioning that by 2020 90% of people living with HIV know their status, 90% of those who know their status are accessing treatment, and 90% of those receiving treatment have durable viral suppression [1]. We estimated the number of people receiving ART in all LMICs under this target using the UNAIDS Fast-Track scenario. We applied the Goals model [20] to 45 countries with a high burden of ART using the Fast-Track coverage targets for all interventions (biomedical prevention, behavioural prevention, and treatment). The Goals model is an HIV simulation model that calculates the spread of HIV as a result of the distribution of risk behaviours in the population and the coverage of prevention and treatment interventions. The model has been used widely for developing HIV strategic plans, investment cases, and Global Fund applications. We then expanded the results to all countries based on the UNAIDS global estimates and projections [19] regarding each country’s contribution to the AIDS epidemic. We assumed that the scale up of current coverage to meet the treatment target by 2020 would be linear, with approximately 26 million people receiving ART by 2021 if all LMICs achieve these targets [20]. This translates to 4.4 million new people receiving ART, and 21.7 million continuing to receive treatment [20].

To estimate the testing demand, we applied WHO guidelines [10] to these numbers for CD4 tests (one test when treatment starts as a baseline, after 6 months, and annual for 10% of people for monitoring treatment failure until patients are stable on treatment and CD4 cell count monitoring can be stopped in individuals who are stable on ART and virally suppressed in settings where routine viral load monitoring is available), VL tests (one test 6 months after treatment starts, at 12 months, and annual thereafter), and EID (an average of 2.12 tests per infant born to an HIV-positive mother to account for tests at birth, six weeks, and/or 9 months). The Fast-Track projection for rapid diagnostic tests assumes that for generalised epidemics and hyper-epidemics, the percentage of the adult population testing annually will increase to 35% by 2020, then decline once the majority of HIV-positive people are identified. However, in countries with heterogeneous epidemics, the coverage target only applies to the highest prevalence areas, which includes two out of three of HIV-positive people. For concentrated epidemics, we made the assumption that there will be annual testing for all sex workers, men who have sex with men, people who inject drugs, transgender people, and prisoners, and 35% annual testing for people with multiple partners or tuberculosis. This assumption was based on that fact that as the percentage of people who know their status increases to the goal of 90% by 2020, testing approaches will need to move from general population testing to focus on population where the yield will be highest, which would be partners of those newly diagnosed and population with the highest incidence (such as key populations and those with TB).

Full country level results can be obtained from the authors, subject to a national consultation process.

## Results

The following table (Table 1) summarises the projected number of CD4 tests, VL and EID tests and rapid diagnostic tests. Each test trend is discussed after the table.

**Table 1:** Summary of projected demand of diagnostic tests.

Type of tests	Type of projection	2016	2017	2018	2019	2020	2021
**CD4 tests**	Fast Track	16.3M	15.3M	13.0M	9.5M	4.8M	2.5M
	Consolidated	17.8M	17.5M	17.0M	16.9M	16.8M	16.6M
							
**VL tests**	Fast Track	18.3M	23.8M	27.7M	30.8M	33.3M	30.8M
	Consolidated	10.7M	14.6M	18.1M	21.6M	24.4M	28.5M
							
**EID tests**	Fast Track	1.6M	1.9M	2.0M	2.2M	2.3M	2.2M
	Consolidated	1.2M	1.4M	1.6M	1.9M	2.0M	2.1M
							
**Rapid diagnostic tests**	Fast Track	390M	417M	441M	462M	480M	437M
	Consolidated	405M	435M	459M	478M	495M	509M

### CD4 cell count tests

**Fig 1:**
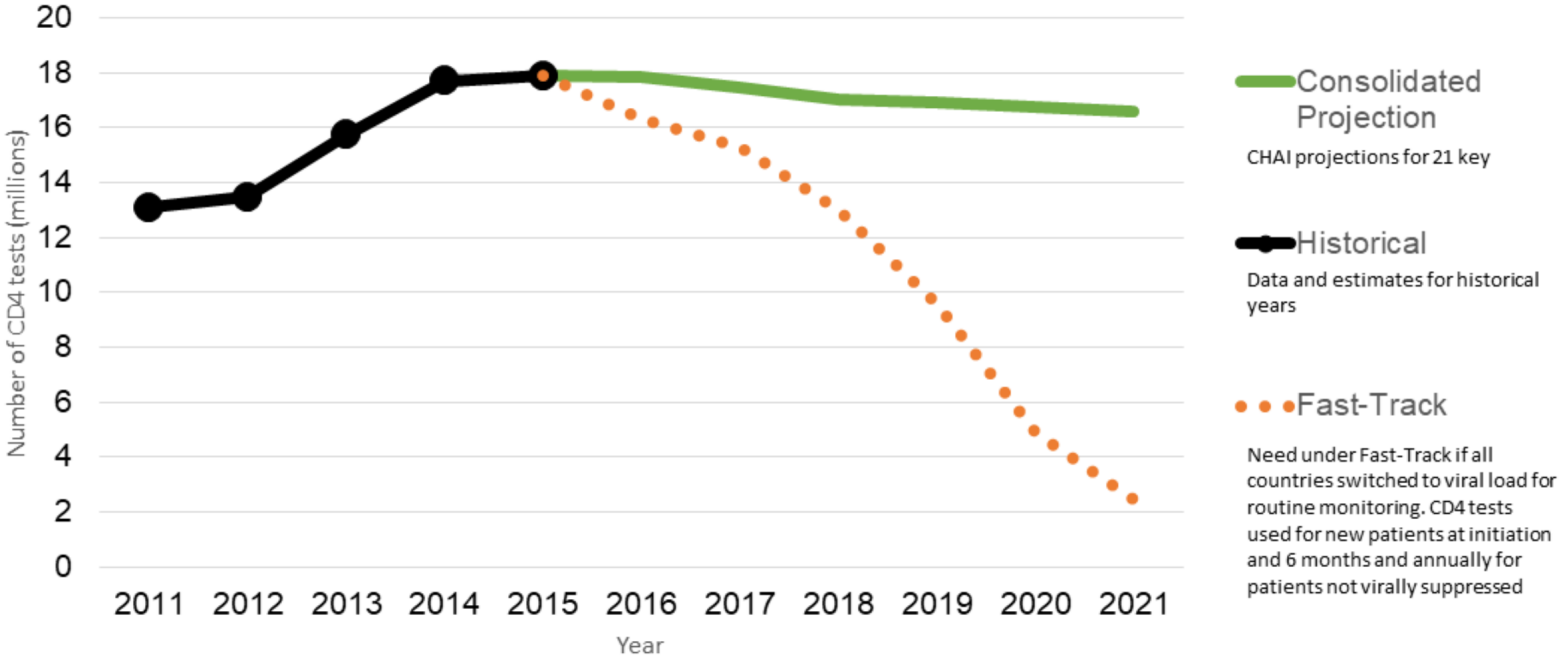
Demand for CD4 count tests, 2011–2021.

Our forecast estimates a gradual decline in demand to 16.6 million in 2021, reflecting the switch to VL monitoring in LMICs. The greatest proportion of these tests are conducted in countries with the highest number of people living with HIV, in particular, South Africa, followed by India, Mozambique, Uganda, Ethiopia, Zambia, and those who perform the highest number of tests per patient on ART such as Brazil. Fig 1 presents our estimates of historical use and projected demand for CD4 cell count tests. The CHAI forecast accounts for approximately 80% of CD4 tests.

### VL tests

**Fig 2:**
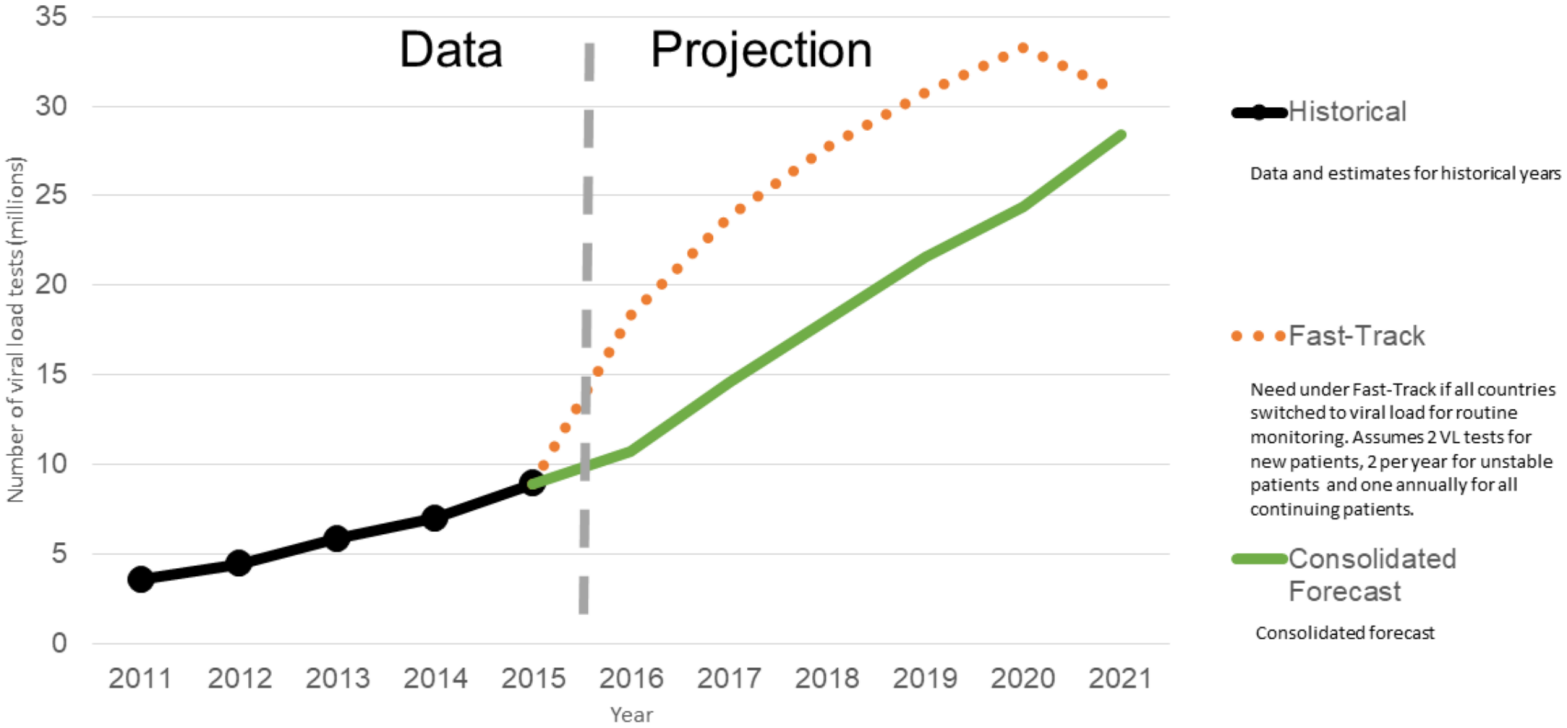
Demand for viral load (VL) tests, 2011–2021.

At the end of 2014, this ratio was 0.57 tests per person receiving ART. We project that demand for VL tests will increase rapidly to approximately 28.5 million in 2021 as the number of people receiving ART increases and more countries adopt VL testing for routine patient monitoring. As for CD4 tests, the largest market for VL tests is South Africa, followed by Brazil, Kenya, Thailand, and Namibia. This list includes countries with high burden (Kenya) and those early adopters of VL tests that have nationwide availability (Brazil, Thailand, Namibia). Fig 2 presents the historical trends and projected demand for VL tests. For VL testing, the CHAI forecast accounts for approximately 85% for the total.

### EID tests

At the end of 2015, the ratio of the number of EID tests to the number of HIV infected pregnant women was1.16 tests. Growth has been slow over the past four years, with demand comprising only 50% of the need. Our forecast for EID tests estimates that countries will increase tests in line with WHO guidelines, with some countries already conducting testing at-birth as well as later on for these infants. We anticipate that demand will increase to 2.1 million tests by 2021. For EID, the CHAI forecast accounts for about 96% of tests, which reflects the Fast-Track estimates, though we estimate growth would be slower than in the more rapid Fast-Track scale-up. Under the Fast-Track scenario, the number of EID tests declines after 2020 because the incidence of HIV infection will decline after 2020 as a result of increased coverage of ARV treatment and impact of prevention efforts. This will lead to fewer HIV infected pregnant women and thus less need for EID tests, which are only used for children born to HIV infected women. For EID POC contribution to scale up of EID tests, we have based our assumptions on the fact that overall EID growth trends will be influenced by POC EID testing that is expected to comprise 5% of the overall EID testing volume in 2016, and 25% by 2020.

**Fig 3:**
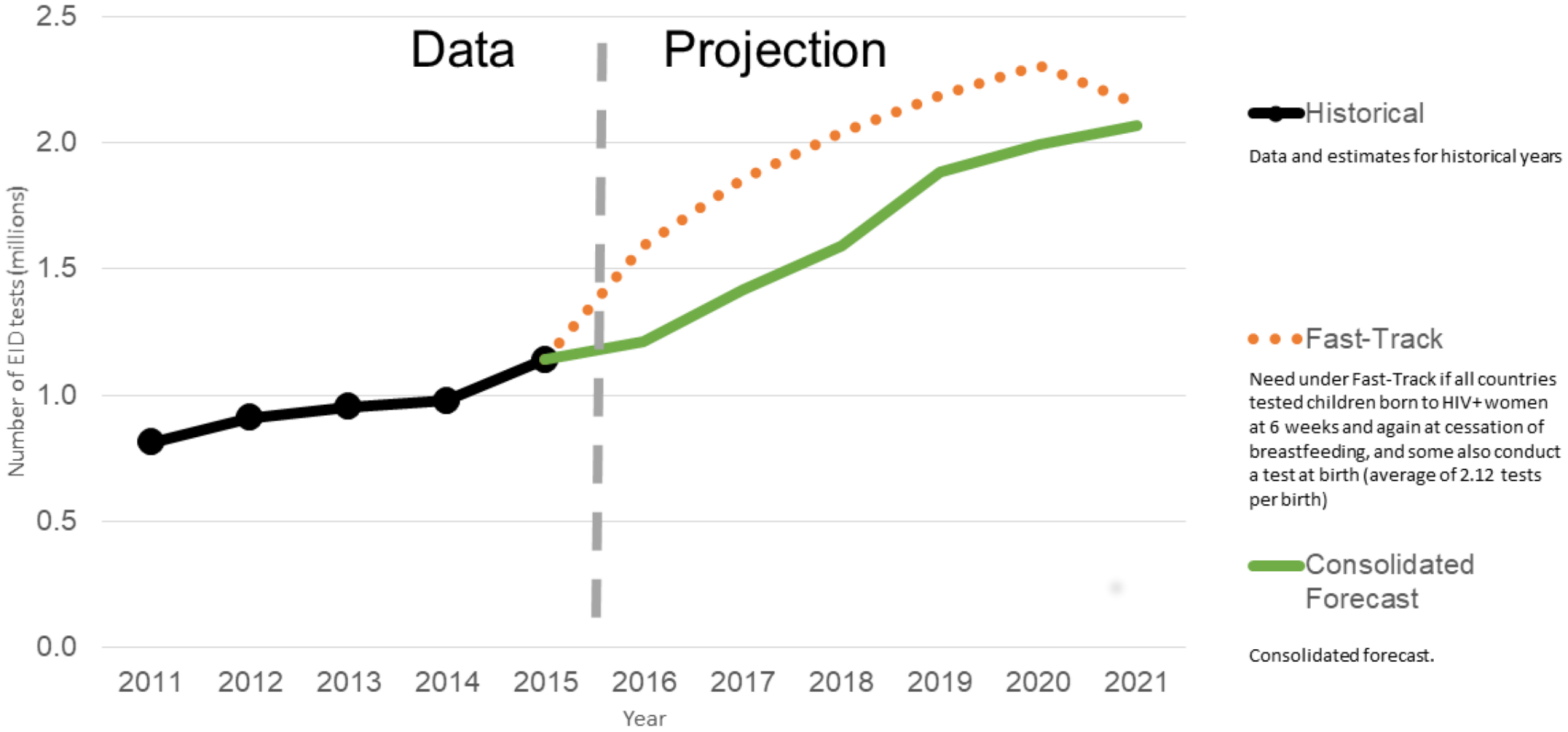
Demand for EID tests, 2011–2021.

In line with CD4 and VL tests, the distribution of reported EID tests in 2015 show the largest market is in South Africa, followed by Uganda, Mozambique, Nigeria, Kenya, and Zimbabwe, which reflect the countries with the highest burden of HIV-positive women. Fig 3 illustrates EID test trends for 2011–2021 and the projected demand.

### Rapid diagnostic testing

**Fig 4:**
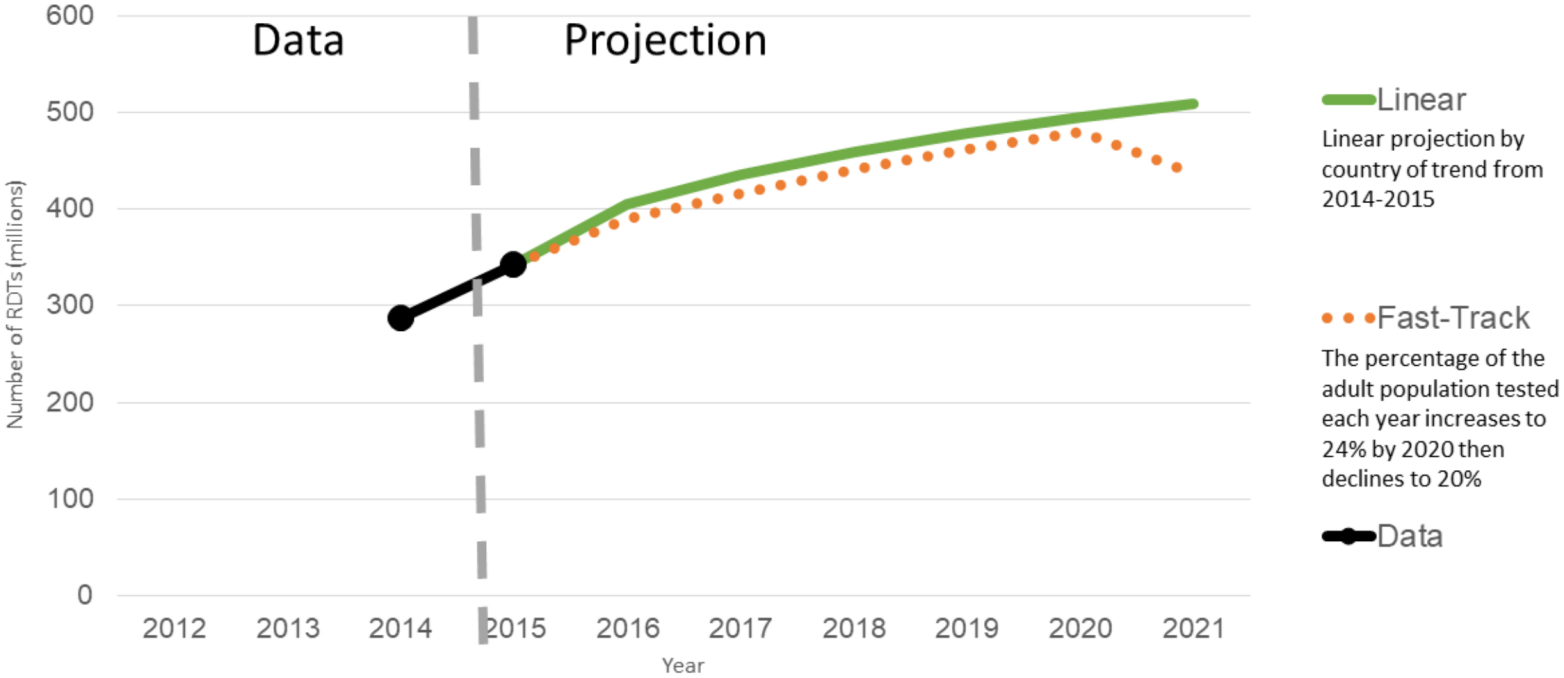
Demand for rapid diagnostic tests, 2011–2021.

Based on the available data on use of rapid diagnostic tests globally in the 2014 and 2015 WHO diagnostic surveys, we estimate that the uptake of RDTs is likely to increase, with demand reaching above 500 million tests by 2021. The largest market for rapid diagnostic tests according to the 2014 WHO survey is China, followed by India, Brazil, Uganda, and Ethiopia. Fig 4 illustrates rapid diagnostic test trends for 2011–2021 and the projected demand.

## Discussion

### Summary of main findings

The findings presented above are the result of the effort of the collaboration of several partners with WHO leadership on this important public health subject. Forecasting the demand of diagnostic and monitoring tests is essential for planning the reagents production and securing the necessary supply and funding to ensure that all people in need are covered.

We forecast that the overall number of CD4 tests is expected to decline between now and 2021 as more countries adopt test-and-treat and shift to VL testing for routine patient monitoring. Our consolidated forecast projects a gradual decline in demand for CD4 tests to 16.6 million by 2021. We anticipate that demand for VL tests will increase rapidly to 28.5 million by 2021, reflecting the increasing number of people who will receive ART and the adoption of VL testing for patient monitoring. We expect that the demand for EID tests will grow more rapidly than in past years, driven by the implementation of testing at birth in programmes globally, in line with WHO guidelines, doubling to 2.1 million tests by 2021. Demand for rapid diagnostic tests is also likely to increase, reaching 509 million tests by 2021.

Testing method or technology used by countries to perform a test was not covered in this forecast. This manuscript reports the coverage and the demand projection of HIV tests used to diagnose the HIV infection, referred to as HIV rapid diagnostic tests (RDTs). We acknowledge the positive impact of POC technologies as POC CD4 increased the coverage in CD4 tests. Likewise, we believe POC EID and POC VL technologies will increase EID and VL coverage. This will be reflected in country and global coverage which will affect the global projections. However, this paper did not attempt to include the technology in the parameter forecast but we are confident that new technologies such as POC EID and POC VL are reflected in our global demand forecast in view of their influence on country coverage and national projections with their ultimate contribution to the global forecast.

### Comparisons with UNAIDS Fast-Track projections

CD4 testing is recommended at baseline for all patients starting treatment, and also to manage advanced HIV disease and opportunistic infections in unstable patients on treatment. Nevertheless, the overall number of CD4 tests is expected to decline as more countries adopt test-and-treat and shift to VL testing for routine patient monitoring. Our consolidated forecast projects a gradual decline in demand for CD4 tests to 16.6 million by 2021, while the Fast Track assumes the transition from CD4 to VL for routine monitoring will happen more rapidly, decreasing to 2.5 million CD4 tests by 2021.

Based on our projections, we anticipate that demand for VL tests will increase rapidly to 28.5 million by 2021. This reflects the increasing number of people who will receive ART and the adoption of VL testing for patient monitoring. The Fast-Track projections assumes that by 2020, all LMICs will have switched to VL to monitor the 26 million people expected to be on ART; this projection shows a more rapid increase, with VL testing volumes reaching 33 million by 2020, dropping to 31 million a year later as fewer new patients need two VL tests.

We expect that demand for EID tests to grow more rapidly than in past years, driven by the implementation of testing at birth as per the WHO guidelines calling for expansion of testing at birth globally. Our forecast estimates that demand may more than double to over 2 million tests by 2021. This estimate is lower than Fast-Track projections, which estimate demand will reach 2.3 million in 2020 before declining after the coverage target of 95% is achieved for prevention of mother-to-child transmission. This aspirational target assumes EID coverage will increase from its current level around 50% to 90% by 2020.

### Limitations

This work represents an attempt to forecast demand for key diagnostics in the field of HIV/AIDS globally, but data must be interpreted with caution. It is important to acknowledge that the projections we present here utilise a range of data sources, including those developed by WHO, UNAIDS, Avenir Health, and CHAI. Projections are also based on data for the number of people receiving ART through to 2016 and projections for use of ART through to 2021, which are based on UNAIDS global reports and Fast-Track targets. Furthermore, our estimates should be interpreted with the understanding that the projections are based on extrapolations of past trends, and key assumptions about future demand, including the achievement of 90-90-90 targets and that countries will expand their programmes in accordance with recent WHO guidelines. Furthermore, for some of our forecasts, our projections may have been limited by the availability of data, for example on the use of rapid diagnostic tests for which there are only two data points from two sources (2014 and 2015 WHO diagnostic Surveys [6,8]), or for market forecasts, which vary based on the state of the market and available data. Further limitations of our model are that it does not account for people who disengage from care and re-present with advanced HIV disease and needing a CD4 cell count to determine level of immunosuppression. A recent study from South Africa found that 25% of patients had disengaged from care within 2 years after starting ART; of these 16% were hospitalised and 3% died [21]. For our EID forecasting, we did not explicitly take into account the introduction of self-testing, which is difficult to predict going forward, but which will undoubtedly impact on test demand to 2021. In order to provide robust projections of demand going forward, there is a continuing need to strengthen reporting and the quality of data globally, particularly in LMICs.

### Next steps for HIV diagnostic tests

In order to achieve the ambitious 90-90-90 targets, it will be essential to improve access to effective and efficient diagnostic tests. This reflects the emphasis on maintaining access to CD4 testing to measure the risk of disease progression and expanding VL testing to assess the efficacy of treatment, EID to identify HIV status in children born to women living with HIV, and rapid diagnostic tests to improve the feasibility and efficiency of HIV diagnosis in LMICs. These projections provide insight into the global demand we can expect to see for these diagnostic tests, both in relation to historical trends, and the UNAIDS Fast-Track targets. The forecasts in this report will inform advocacy for improving access to diagnostics in order to achieve the UNAIDS 90-90-90 targets for HIV treatment, and ensure global players meet the anticipated demand.

The projected trends in demand for HIV diagnostic tests in LMICs will be influenced by efforts to reduce visit and diagnostic monitoring frequency, shift monitoring from CD4 to VL testing, adoption of the most recent paediatric guidelines, and make testing more efficient by adopting and implementing WHO HIV testing guidelines. Furthermore, increasing availability of point-of-care technologies for VL, and EID tests is likely to contribute to demand, playing a particularly important role in strengthening diagnostic capacity in the context of decentralisation of HIV care, which may present challenges to meeting the targets in the hardest hit countries without these technologies [6,22,23]. However, it is uncertain how this will influence the projections described in this paper. Additional factors to consider which may impact on achievement of diagnostic targets include the availability of funding, economic development, and the prevalence of HIV and other infections like TB and viral hepatitis, which will contribute to the demand for similar diagnostic approaches [6].

While successful scaling-up of ART in LMICs has facilitated the treatment of 19.5 million people, our projections in this report are valuable in supporting programme managers and funding agencies to scale up and utilise these tests to meet the demand, and serve to inform advocacy for improving access to diagnostics to achieve the ambitious 90-90-90 targets. In doing so, it will be important to take into account available evidence of potential barriers to achieving these targets, including financial constraints and other key factors that are known to contribute to incomplete or slow implementation of the latest recommendations to Treat All [24].

These findings highlight the ongoing need for governments and international funding bodies to prioritise improving capacity and access to HIV diagnostic and monitoring technologies in line with the demand we have projected here. This will be essential for achieving the UNAIDS 90-90-90 target for HIV diagnosis and treatment, and meeting our obligation, both to the 36.7 million people who are living with HIV as well as to those to whom we have a continuing commitment to improve access to high-quality testing and treatment [1].

## Supporting information

S1 File WHO survey and other country data. (XLSX)

S2 File CHAI country diagnostic test data. (XLSX)

S3 File Consolidated and Fast-Track diagnostic test data. (XLSX)
